# Regional disparities in health literacy for chronic diseases: focusing on healthcare resources and local extinction index

**DOI:** 10.3389/fpubh.2024.1423645

**Published:** 2024-09-12

**Authors:** Seokmin Ji, Young Gyu Kwon, Hyunseo Lee, Chaehwan Shin, Minsung Sohn, Mankyu Choi

**Affiliations:** ^1^Department of Health Policy & Management, College of Public Health Science, Korea University, Seoul, Republic of Korea; ^2^BK21 FOUR R&E Center for Learning Health Systems, Korea University, Seoul, Republic of Korea; ^3^Center for Medical Education, College of Medicine, Chung-Ang University, Seoul, Republic of Korea; ^4^Division of Health and Medical Sciences, The Cyber University of Korea, Seoul, Republic of Korea

**Keywords:** spatial analysis, propensity score matching, health literacy, regional disparity, local extinction index, South Korea

## Abstract

**Objective:**

This study compared disparities between community health characteristics and health literacy levels for hypertension and diabetes by combining community-level characteristics, such as the local extinction index and healthcare resources, with individual-level characteristics based on the Andersen healthcare utilization model.

**Method:**

Data obtained from the 2017, 2019, and 2021 Community Health Surveys, Korean Statistical Information Service, and National Health Insurance Service were analyzed. The analyses included spatial analysis, propensity score matching, and cross-analysis.

**Results:**

Twenty-five extinction-risk regions (ERRs) were identified in 2017, 26 in 2019, and 29 in 2021, indicating a high risk of extinction and insufficient healthcare resources in non-metropolitan regions. Based on analyses of demographic changes and unmet medical needs at the individual level, we observed increased age and economic activity, decreased healthcare access, and lower education levels in ERRs compared to non-extinction-risk regions (NERRs). No significant differences were found between the regions regarding diagnosis or medication use concerning the health literacy gap for hypertension and diabetes. However, individuals in ERRs were significantly less likely than those in NERRs to be aware of such diseases or educated about their management.

**Discussion:**

Given that healthcare services in ERRs focus on chronic disease management rather than prevention, we propose two directions to reduce health disparities in ERRs. First, the government should encourage cooperation with private healthcare organizations to ensure the provision of health education programs in vulnerable areas. Second, improvements in awareness and education regarding chronic disease management can be achieved through digital healthcare and telemedicine. This study identifies regional disparities in chronic disease prevention and management, providing a basis for policies to ensure healthier communities with health equity.

## 1 Introduction

Declining birthrates and aging populations, especially in developed countries, have resulted in major changes globally. Paying attention to rural and regional population decline, the Organization for Economic Co-operation and Development (OECD) has emphasized the development and implementation of regional development policies (1). Among OECD member countries, South Korea, which is currently experiencing severe demographic changes and regional decline, experienced the dead cross phenomenon in 2020, wherein deaths surpassed births. Additionally, it is predicted that South Korea will become a super-aged society by 2025, which is significantly sooner than other OECD countries (2, 3).

Rapid demographic changes carry a significant risk of rural depopulation and a subsequent decline in healthcare resources. Depopulation, also known as population decline, is the overall reduction or extinction of a region’s population. It can significantly affect a country’s long-term socioeconomic sustainability, and must therefore be managed effectively (4–7). Population decline in extinction-risk regions (ERRs) results in the closure of existing medical institutions due to the shortage of medical personnel. Korea’s healthcare system is predominantly reliant on private providers, and the shutdown of medical institutions has reduced access to local healthcare services, thereby resulting in notable systemic gaps (8, 9).

Declining healthcare resources resulting from population decline exacerbate the loss of health equity among individuals with chronic diseases across various regions globally. The achievement of health equity among individuals with chronic diseases such as hypertension, diabetes, and cardiovascular diseases is closely linked to the availability of healthcare resources, since such diseases are characterized by individual health behaviors as well as their physical and socioeconomic environments (10, 11). In such environments, the concept of health literacy is emerging, with the key aim of helping individuals maintain and improve their health despite the presence of chronic diseases (12–14). Health literacy encompasses individuals’ cognitive and social skills, which reflect their motivation and capacity to comprehend health-related information. It also involves their aptitude to apply this information in managing personal health using available healthcare services. Health literacy positively impacts patient safety, access to healthcare, and quality of care, and is increasingly crucial in the enhancement of health equity in different regions (15, 16).

To date, in various ERRs, studies on health equity among individuals suffering from chronic diseases have been conducted at the community and individual levels. At the community level, the related studies simply examine and visualize the changes in spatial distribution (17–20). However, at the individual level, the studies primarily focus on the characteristics of individuals with chronic diseases and their perceived health problems as individual responsibilities (21–23). Therefore, this study aimed to identify regional health disparities in health literacy for chronic diseases by combining community-and individual-level characteristics to provide policy directions to resolve health disparities.

First, employing the local extinction index (LEI) along with hospital and clinic counts, we assessed population changes related to healthcare resources. Based on this assessment, we categorized regions as ERRs and non-extinction-risk regions (NERRs). Second, we identified the regions’ health characteristics by comparing demographic characteristics, such as gender, age, education levels, and unmet medical needs, between ERRs and NERRs. Third, we compared the levels of health literacy for hypertension and diabetes between ERRs and NERRs. Finally, we suggested policy directions for addressing health disparities between these regions. This study seeks to provide an empirical basis for policy formulation aimed at addressing regional disparities in chronic disease prevention and management, while ensuring the achievement of community health promotion that considers health equity among all individuals in the regions involved.

## 2 Materials and methods

### 2.1 Local extinction index

This study uses the local extinction index to measure the regional decline potential among women in the reproductive age group of 20–39 years. A significant reduction in this group can indicate a region “at risk” due to challenges in social security and employment (24). Lee expanded on Masuda’s method by examining the ratio of women aged 20–39 to those aged 65 and older. An index of 1.5 or higher indicates a “Very low risk of extinction,” 1.0–1.5 signifies a “Moderate risk of extinction,” 0.5–1.0 suggests “Caution stage,” 0.2–0.5 indicates “Entering the risk of extinction,” and below 0.2 denotes “high risk of extinction” (24). The formula used is:


$$\text{Local extinction index}=\frac{\text{Women Aged}\;20-39\;\text{Years}}{\text{Population Aged}\;65\;\text{Years and Above}}$$

This index is essential for understanding regional disparities in healthcare resources and health literacy. The primary goal is to offer empirical evidence that supports targeted policy interventions and resource allocation to mitigate healthcare inequities in high-risk regions, thereby improving overall health outcomes.

### 2.2 Study area

**Figure 1:**
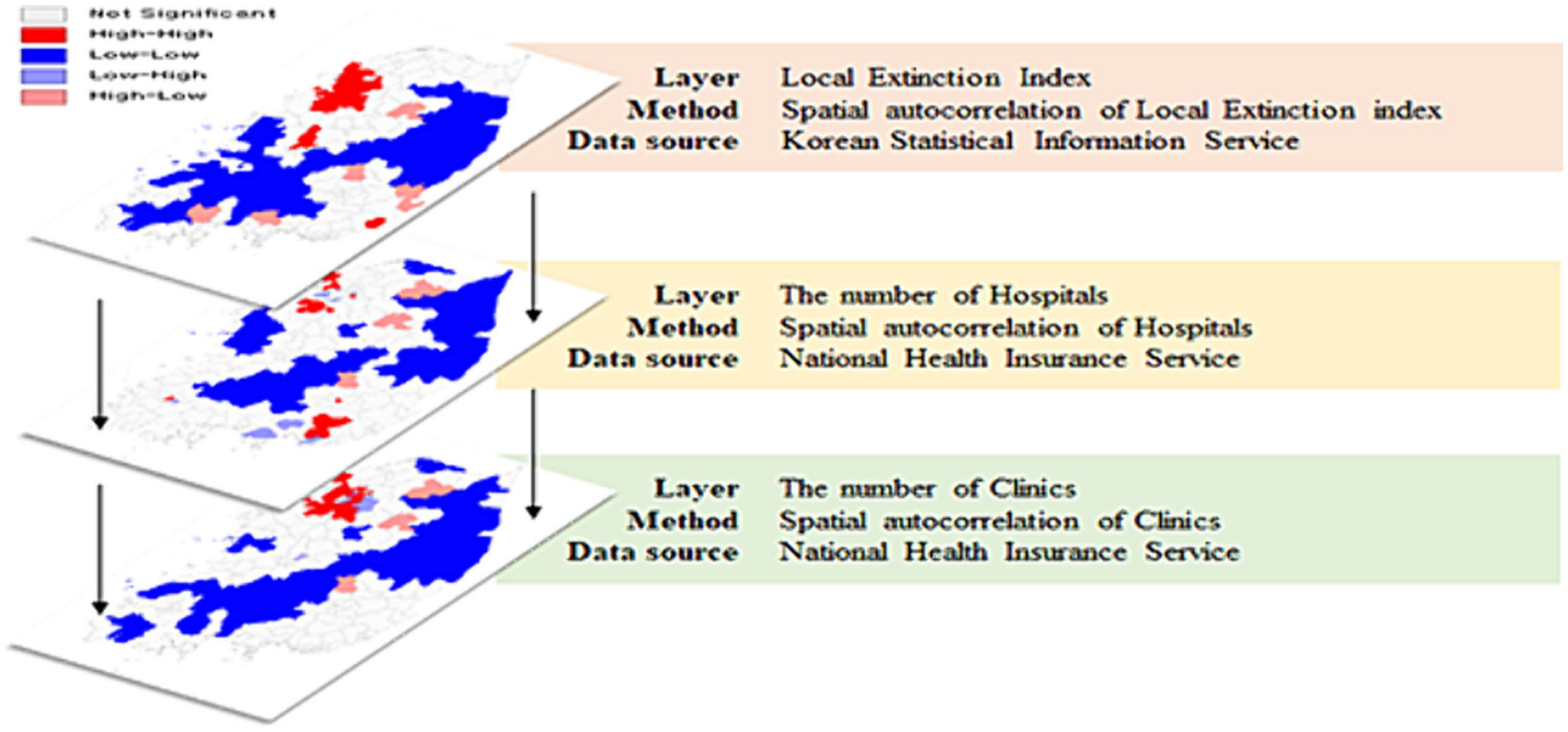
Steps of identifying the study area.

In this study, we compared the levels of health literacy among individuals suffering from chronic diseases by categorizing regions into ERRs and NERRs based on the LEI and healthcare resource status, as presented in Figure 1. Regions with a low LEI typically have fewer healthcare facilities due to population decline, thereby hindering access to healthcare and timely symptom management (3). Conversely, areas with a high LEI enjoy improved access to healthcare due to population growth or revitalization, thereby promoting the early detection and treatment of chronic diseases (25). Consequently, ERRs include regions with a low LEI and limited healthcare availability, whereas NERRs encompass areas with a high LEI and abundant medical services.

### 2.3 Data sources and definition of variables

This study utilized both community-and individual-level data for analysis. Geoda (ver. 1.20.0.10) and QGIS (ver. 3.24.1) were employed for the spatial analysis and geographical pattern visualization of the community-level data. The STATA software (ver. SE. 17) was used for the statistical analysis of individual-level data. The detailed definitions of the variables involved are listed in Table 1.

**Table 1:** Variable definitions.

Variables		Definition of variables	Source
Community based data	Local extinction index	The numerator of the regional extinction index is defined as women between the ages of 20 and 39 years, and the denominator is the population of individuals aged 65 years and above	Korean Statistical Information Service (2017, 2019, 2021)
	Number of hospitals	The number of hospitals at the regional level	National Health Insurance Service (2017, 2019, 2021)
	Number of clinics	The number of clinics at the regional level	
Individual based data	Gender	Men, Women	Community Health Survey (2017, 2019, 2021)
	Age	Continuous	
	Education level	Under elementary school, Middle school, High school, College or Higher	
	Spouse or not	Yes, No	
	Economic activity	Yes, No	
	Unmet medical needs	Yes, No	
	Diagnosis	Diagnosis for hypertension/diabetes (Yes, No)	
	Medication	Medication for hypertension/diabetes (Yes, No)	
	Awareness	Awareness of blood pressure/glycemic (Yes, No)	
	Education	Education of hypertension/diabetes management (Yes, No)	

Community-level data were obtained from the Korean Statistical Information Service and National Health Insurance Service, spanning 2017, 2019, and 2021. The LEI was calculated by dividing the number of women aged 20–39 years by the number of individuals aged 65 years and older within a region. A lower LEI reflects reduced fertility rates and increased aging, which correlates with a heightened risk of local extinction. The number of hospitals and clinics was determined by the number of medical facilities in the area.

Individual-level data were obtained from the 2017, 2019, and 2021 Community Health Surveys. These data are actively used in research to evaluate health at the community level because they contain information regarding health behaviors, morbidity rates, and the utilization of healthcare services among local residents, with the main aim of implementing evidence-based health projects required in each region (26, 27). Therefore, this study used gender, age, education, and spousal status as the predisposing factors, economic activity as a probability factor, and unmet medical needs as a need factor to identify the characteristics of healthcare-service utilization according to the Andersen healthcare utilization model (28). Additionally, in this study, health literacy was defined as the ability of individuals to understand, access, and utilize health information to maintain and improve their health (29, 30). Therefore, to evaluate and compare the levels of health literacy for hypertension and diabetes by region, the following four dependent variables were used: diagnosis, medication, awareness, and management education.

### 2.4 Spatial autocorrelation

Spatial data refers to the interdependencies and interactions between geographical regions and spaces that exhibit similar characteristics, which are characterized by the fact that the closer they are spatially, the more similar and correlated they are (31). In this study, we used Moran’s I statistic presented by Moran (32). The basic formula is shown in Equation 1, as follows:


(1) $$\text{Mora}n^{\prime }sI=\frac{N\sum_{i=1}^{n}\sum_{j=1}^{n}\omega_{ij}\left(Y_{i}-\bar{Y}\right)\left(Y_{j}-\bar{Y}\right)}{\left(\sum_{i=1}^{n}\sum_{j=1}^{n}\omega_{ij}\right)\sum_{i=1}^{n}{\left(Y_{i}-\bar{Y}\right)}^{2}}$$

where *N* represents the number of regional units, *Y* represents the dependent variable, *Y_i_* represents the mean of *Y* in region *i*, and *ω_ij_* represents the spatially weighted matrix of points *i* and *j*. Moran’s *I* index uses a Z-test to determine statistical significance, and the basic formula is shown in Equation 2, as follows:


(2) $$Z=\frac{I-E\left(I\right)}{S_{e}\left(I\right)}$$

where E(I) and $S_{e}\left(I\right)$ represent the mean and standard deviation of the statistic I. The spatial autocorrelation index of Moran’s *I* has values ranging from −1 to 1, with values closer to 1 indicating a positive correlation and values closer to −1 indicating a negative correlation (33).

### 2.5 Propensity score matching

Propensity score matching is a sampling technique that pairs samples with similar characteristics to ensure homogeneity between groups, and is used to narrow the gap between control and treatment groups based on the observed covariates (34, 35). The probit regression used in the analysis is shown in Equation 3.

This probit regression implies a linear transformation of the nonlinearity of the bivariate dependent variable based on the principle of binomial distribution, which states that the mean *EY* of the bivariate dependent variable is equal to the probability *p* of performing a certain action and the properties of the normal distribution function (36).


(3) $$\text{probit}\left(EY\right)=\Phi^{-1}\left(p\right)=\Phi^{-1}\left(P\left[Y=1\right]\right)=\beta_{0}+\sum_{j=1}^{k}\beta_{j}x_{j}$$

The process comprises three steps: estimating the propensity scores, matching between groups, and evaluating the quality of the matches (36). First, the group variable, which distinguishes between the control group (ERRs) and the treatment group (NERRs), was selected according to the LEI, number of hospitals, and number of clinics and set as the dependent variable. We used gender, age, education, spousal status, economic activity, and unmet medical needs as the independent variables, which, according to the Andersen model, can affect the utilization of healthcare services. The propensity scores were then estimated using probit regression.

**Figure 2:**
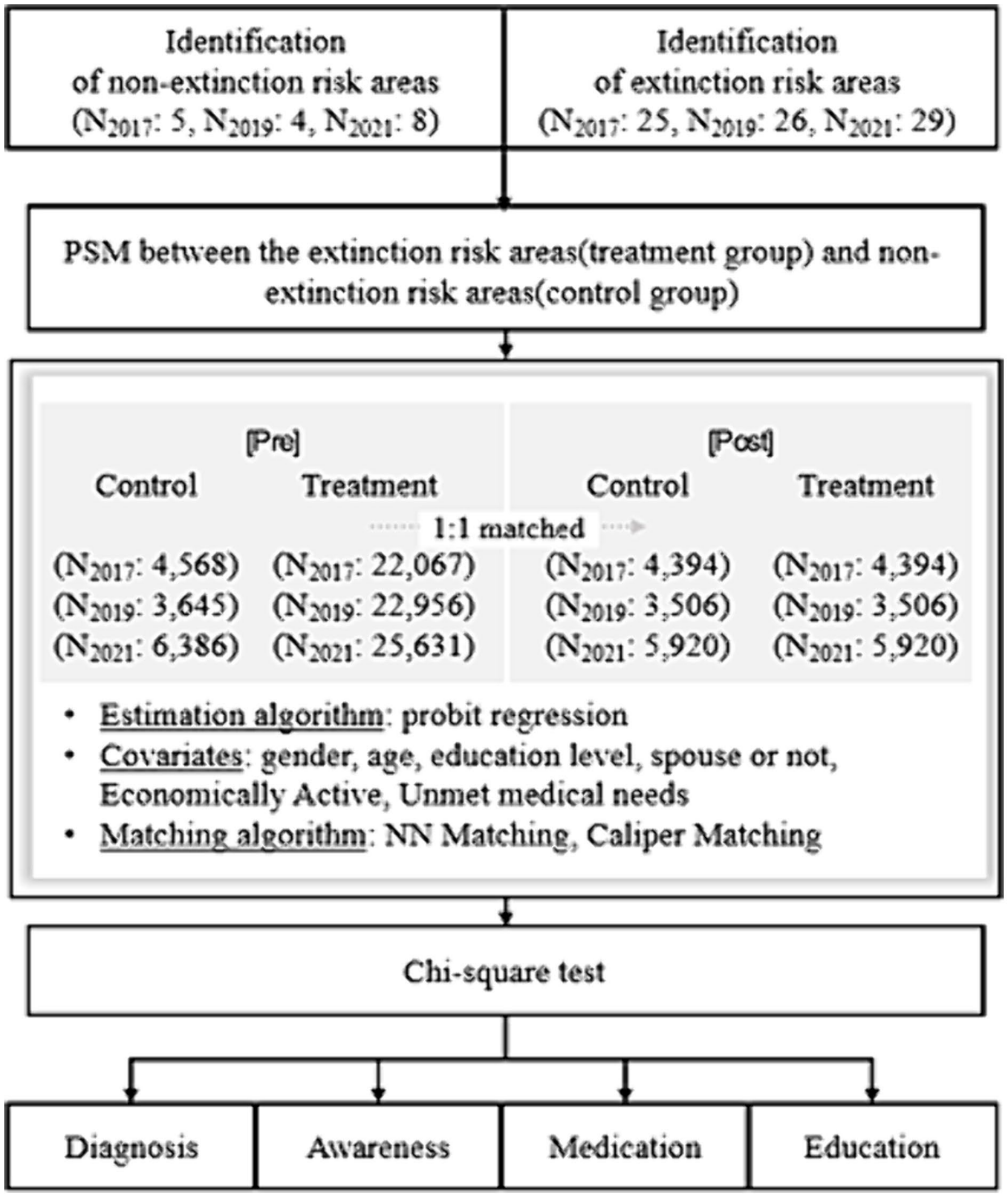
Research model.

Second, we used the 1:1 NN matching method, which is efficient when the control group is large and has the advantage of fewer discarded observations. We also used the 0.001 caliper matching method, which can adjust for differences in propensity scores between matched samples (37). Third, to assess the matching quality, we used the balancing test method, which tests the distributional shape of covariates in the treatment and control groups before and after matching. In this study, we confirmed that the histogram distributions of the propensity scores for both groups matched (Supplementary Appendix Table A1). The flowchart for this study is presented in Figure 2.

### 2.6 Ethical statement

The Korea University Institutional Review Board reviewed and approved this study (approval number: KUIRB-2023-0242-01).

## 3 Results

### 3.1 Identifying the study area

**Figure 3:**
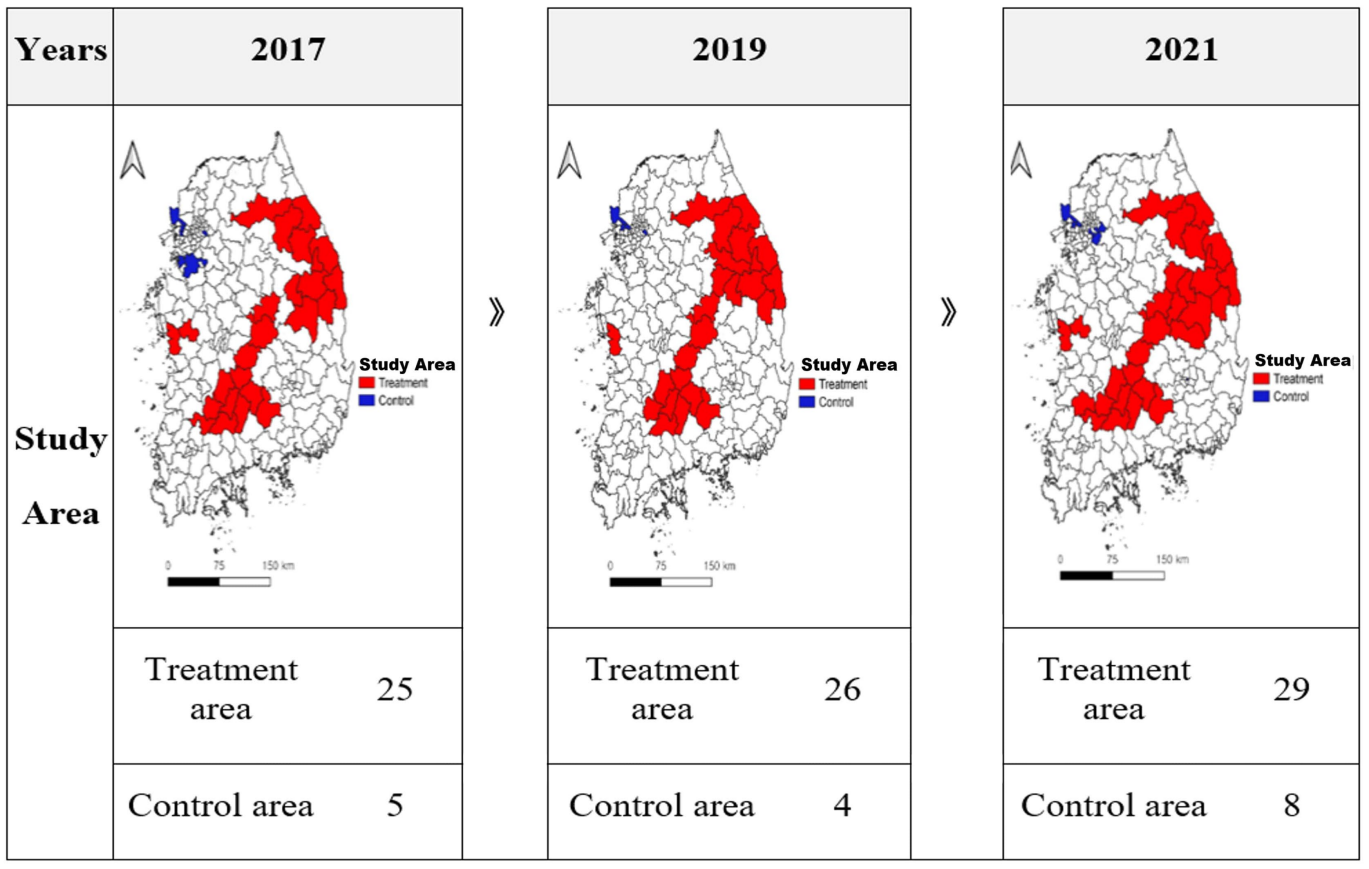
Study area.

Table 2 shows the number of regions identified through spatial autocorrelation analysis based on the LEI, number of hospitals, and number of clinics during the study period. In 2017, 2019, and 2021, the ERRs of LEI were 52, 53, and 53 regions, and the NERRs were 49, 50, and 49 regions, respectively. In 2017, 2019, and 2021, the ERRs of number of hospitals were 12, 12, and 15 regions, and NERRs were 37, 39, and 41 regions. In 2017, 2019, and 2021, the ERRs of number of clinics were 29, 34, and 32 regions, and the NERRs were 44, 44, and 45 regions. Figure 3 shows the final regions for this study. Finally, 25, 26, and 29 ERR regions were selected as the target regions in 2017, 2019, and 2021, and 5, 4, and 8 NERR regions were selected (Supplementary Appendix Table A2).

**Table 2:** Number of regions for community based data.

	2017		2019		2021	
	ERR	NERR	ERR	NERR	ERR	NERR
LEI	52	49	53	50	52	49
Hospitals	37	12	39	12	41	15
Clinics	44	29	44	34	45	32

LEI, local extinction index; ERR, extinction risk regions; NERR, non-extinction risk regions.

### 3.2 Results of propensity score matching

The results before and after propensity matching for each year throughout the study period are listed in Table 3. In 2017, the number of participants in the treatment and control groups before propensity score matching were 22,067 and 4,568, respectively, which was adjusted to 4,394 after propensity score matching. In 2019, the numbers of participants in the treatment and control groups before propensity score matching were 22,956 and 3,645, respectively, which was adjusted to 3,506 after propensity score matching. In 2021, the number of participants in the treatment and control groups before propensity score matching were 25,631 and 6,386, respectively, which was adjusted to 5,920 after propensity score matching. *T*-tests for the mean differences in the variables used for propensity score matching and cross-tabulation analysis revealed significant differences between the treatment and control groups in terms of age, spousal status, and economic activity prior to propensity score matching for all 3 years. We also found significant differences in the responses to unmet medical needs in 2017 and 2021. However, after propensity score matching, we found no significant differences between the treatment and control groups for most variables in 2017, and for all variables in 2019 and 2021. Therefore, homogeneity between the two groups was achieved, and the characteristics of each variable were similar.

**Table 3:** Descriptive statistics.

		Before matching, *N* (%)								
		2017			2019			2021		
		Control	Treatment	*P*	Control	Treatment	*P*	Control	Treatment	*P*
		4,568	22,067		3,645	22,956		6,386	25,631	
		(17.15)	(82.85)		(13.70)	(86.30)		(19.95)	(80.05)	
Gender	Men	2,063	9,768	0.267	1,601	10,223	0.491	2,905	11,631	0.337
		(45.16)	(44.27)		(43.92)	(44.53)		(45.49)	(45.38)	
	Women	2,505	12,299		2,044	12,733		3,481	14,000	
		(54.84)	(55.73)		(56.08)	(55.47)		(54.51)	(54.62)	
Age (years)	Continuous (Mean)	47.91	59.96	0.000	50.30	61.44	0.000	49.75	61.25	0.668
Education level	≤Elementary school	460	9,316	0.000	387	9,575	0.000	481	9,553	0.195
		(10.07)	(42.22)		(10.62)	(41.71)		(7.54)	(37.27)	
	Middle school	387	2,855		312	3,103		430	3,473	
		(8.47)	(12.94)		(8.56)	(13.52)		(6.73)	(13.55)	
	High school	1,364	5,123		1,060	5,529		1,665	6,533	
		(29.86)	(23.22)		(29.08)	(24.09)		(26.07)	(25.49)	
	≥College	2,357	4,773		1,886	4,749		3,810	6,072	
		(51.60)	(21.63)		(51.74)	(20.69)		(59.66)	(23.69)	
Spouse or not	Yes	3,037	15,217	0.001	2,361	15,715	0.000	3,935	16,702	1.000
		(66.48)	(68.96)		(64.77)	(68.46)		(61.62)	(65.16)	
	No	1,531	6,850		1,284	7,241		2,451	8,929	
		(33.52)	(31.04)		(35.23)	(31.54)		(38.38)	(34.84)	
Economic activity	Yes	2,799	14,345	0.000	2,145	14,820	0.000	3,942	16,937	0.328
		(61.27)	(65.01)		(58.85)	(64.56)		(61.73)	(66.08)	
	No	1769	7,722		1,500	8,136		2,444	8,694	
		(38.73)	(34.99)		(41.15)	(35.44)		(38.27)	(33.92)	
Unmet medical needs	Yes	455	2,594	0.001	222	1,421	0.002	308	1,496	0.130
		(9.96)	(11.76)		(6.09)	(6.19)		(4.82)	(5.84)	
	No	4,113	19,473		3,423	21,533		6,078	24,135	
		(90.04)	(88.24)		(93.91)	(93.81)		(95.18)	(94.16)	

		After matching, *N* (%)								
		2017			2019			2021		
		Control	Treatment	*P*	Control	Treatment	*P*	Control	Treatment	*P*
		4,394	4,394		3,506	3,506		5,920	5,920	
		(50.00)	(50.00)		(50.00)	(50.00)		(50.00)	(50.00)	
Gender	Men	2,023	1,937	0.065	1,569	1,609	0.337	2,807	2,708	0.068
		(46.04)	(44.08)		(44.75)	(45.89)		(47.42)	(45.74)	
	Women	2,371	2,457		1,937	1,897		3,113	3,212	
		(53.96)	(55.92)		(55.25)	(54.11)		(52.58)	(54.26)	
Age (years)	Continuous (Mean)	47.87	47.99	0.736	50.06	49.89	0.668	49.94	50.34	0.198
Education level	≤Elementary school	460	541	0.001	384	344	0.195	481	492	0.969
		(10.47)	(12.31)		(10.95)	(9.82)		(8.13)	(8.31)	
	Middle school	363	282		299	339		414	404	
		(8.26)	(6.42)		(8.54)	(9.67)		(6.99)	(6.83)	
	High school	1,326	1,330		1,026	1,026		1,613	1,611	
		(30.18)	(30.27)		(29.26)	(29.26)		(27.25)	(27.21)	
	≥College	2,245	2,241		1,797	1,797		3,412	3,413	
		(51.09)	(51.00)		(51.25)	(51.25)		(57.64)	(57.65)	
Spouse or not	Yes	2,960	2,964	0.927	2,318	2,318	1.000	3,676	3,715	0.459
		(67.36)	(67.46)		(66.12)	(66.12)		(62.09)	(62.75)	
	No	1,434	1,430		1,188	1,188		2,244	2,205	
		(32.64)	(32.54)		(33.88)	(33.88)		(37.91)	(37.25)	
Economic Activity	Yes	2,749	2,661	0.054	2,110	2,150	0.328	3,794	3,783	0.833
		(62.56)	(60.56)		(60.18)	(61.32)		(64.09)	(63.90)	
	No	1,645	1,733		1,396	1,356		2,126	2,137	
		(37.44)	(39.44)		(39.82)	(38.68)		(35.91)	(36.1)	
Unmet medical needs	Yes	441	538	0.001	209	180	0.130	283	324	0.088
		(10.04)	(12.24)		(5.96)	(5.13)		(4.78)	(5.47)	
	No	3,953	3,856		3,297	3,326		5,637	5,596	
		(89.96)	(87.76)		(94.04)	(94.87)		(95.22)	(94.53)	

### 3.3 Descriptive statistics

The basic statistics for the two regions by year during the study period are listed in Table 3. Regarding gender, the proportion of women was higher than that of men in both regions throughout the 3 years. In addition, the average age, proportion of people with spouses, and proportion of economically active people were higher among individuals in ERRs compared with those in NERRs. However, for individuals with unmet medical needs and those with low education levels, access to healthcare was lower in ERRs than in NERRs. Specifically, in both regions, the proportion of women was 10.4% higher than that of men on average over the three-year period, and age was 11.6 years older on average during the 3-year period in ERRs than in NERRs. On average, the percentage of people with a college degree or higher was 32.3% lower in ERRs than in NERRs, the proportion of people with a spouse was 3.2% higher in ERRs than in NERRs, the proportion of people who were economically active was 4.6% higher in ERRs than in NERRs, and the proportion of people with unmet medical needs was 1% higher in ERRs than in NERRs.

### 3.4 Health literacy in hypertension across the study area

Table 4 shows the results of a cross-analysis between the ERRs and NERRs to identify health literacy differences for hypertension throughout the study period. In 2021, the analysis showed that ERRs had higher rates of hypertension diagnoses, medication use, and blood pressure awareness, and lower rates of hypertension management education than NERRs. All these differences were statistically significant, and the breakdown by year was as follows.

**Table 4:** Health literacy in hypertension across the study area.

		2017, *N* (%)			2019, *N* (%)			2021, *N* (%)		
		Yes	No	*P*	Yes	No	*P*	Yes	No	*P*
Diagnosis	Control	929	3,465	0.451	786	2,720	0.149	1,288	4,632	0.001
		(21.14)	(78.86)		(22.42)	(77.58)		(21.76)	(78.24)	
	Treatment	958	3,436		837	2,669		1,445	4,475	
		(21.8)	(78.2)		(23.87)	(76.13)		(24.41)	(75.59)	
Medication	Control	821	3,573	0.348	737	2,769	0.316	1,189	4,731	0.000
		(18.68)	(81.32)		(21.02)	(78.98)		(20.08)	(79.92)	
	Treatment	858	3,536		780	2,726		1,372	4,548	
		(19.53)	(80.47)		(22.25)	(77.75)		(23.18)	(76.82)	
Awareness	Control	2,914	1,480	0.000	2,157	1,349	0.000	3,887	2,033	0.000
		(66.32)	(33.68)		(61.52)	(38.48)		(65.66)	(34.34)	
	Treatment	2,599	1,795		2,348	1,158		3,951	1,969	
		(59.15)	(40.85)		(66.97)	(33.03)		(66.74)	(33.26)	
Education	Control	421	3,973	0.000	130	3,376	0.000	462	5,458	0.000
		(9.58)	(90.42)		(3.71)	(96.29)		(7.8)	(92.2)	
	Treatment	179	4,215		81	3,425		239	5,681	
		(4.07)	(95.93)		(2.31)	(97.69)		(4.04)	(95.96)	

First, for hypertension diagnoses, there was a similar trend between ERRs and NERRs in 2017 and 2019. However, in 2021, the difference between the two regions widened, with a higher rate of diagnoses in ERRs than in NERRs. The difference was statistically significant only for 2021. Second, in terms of medication use for hypertension, there was a similar trend between ERRs and NERRs in 2017 and 2019. However, in 2021, the difference between the two regions widened, with a higher rate of medication use for hypertension in ERRs than in NERRs. This difference was statistically significant only for 2021. Third, in 2017, regarding blood pressure awareness, NERRs had a higher awareness rate than ERRs. While the trend reversed in 2019 with the ERR awareness rate increasing, the awareness difference between the two regions narrowed in 2021 to approximately 1%, owing to an increase in awareness in NERRs. Statistically significant differences were observed across all the years. Fourth, in terms of the completion of hypertension management education, NERRs had higher completion rates than ERRs in 2017, 2019, and 2021. In particular, the difference in completion rates between the two regions was high in 2021, with a difference of approximately 20%. Statistically significant differences were observed across all the years.

### 3.5 Health Literacy in Diabetes Mellitus across the study area

Table 5 shows the results of the cross-analysis between ERRs and NERRs to identify the health literacy differences for diabetes during the study period. In 2021, the analysis showed that ERRs had lower rates of glycemic awareness and diabetes management education than NERRs. These differences were statistically significant. However, there were no statistically significant differences in diabetes medication use and diagnosis rates between the two regions. The breakdown by year is as follows.

**Table 5:** Health literacy in diabetes mellitus across the study area.

		2017, *N* (%)			2019, *N* (%)			2021, *N* (%)		
		Yes	No	*P*	Yes	No	*P*	Yes	No	*P*
Diagnosis	Control	403	3,991	0.162	305	3,201	0.210	585	5,335	0.345
		(9.17)	(90.83)		(8.70)	(91.30)		(9.88)	(90.12)	
	Treatment	366	4,028		348	3,158		616	5,304	
		(8.33)	(91.67)		(9.93)	(90.07)		(10.41)	(89.59)	
Medication	Control	352	4,042	0.553	286	3,220	0.348	518	5,402	0.166
		(8.01)	(91.99)		(8.16)	(91.84)		(8.75)	(91.25)	
	Treatment	329	4,065		329	3,177		563	5,357	
		(7.49)	(92.51)		(9.38)	(90.62)		(9.51)	(90.49)	
Awareness	Control	1,070	3,324	0.000	923	2,583	0.276	1,971	3,949	0.000
		(24.35)	(75.65)		(26.33)	(73.67)		(33.29)	(66.71)	
	Treatment	896	3,498		949	2,557		1,970	3,950	
		(20.39)	(79.61)		(27.07)	(72.93)		(33.28)	(66.72)	
Education	Control	188	4,206	0.000	77	3,429	0.000	274	5,646	0.000
		(4.28)	(95.72)		(2.20)	(97.80)		(4.63)	(95.37)	
	Treatment	94	4,300		39	3,467		170	5,750	
		(2.14)	(97.86)		(1.11)	(98.89)		(2.87)	(97.13)	

First, the diagnoses for diabetes were similar between the two regions in 2017, 2019, and 2021. There were no statistically significant differences between the years. Second, the use of diabetes medication was similar between the two regions in 2017, 2019, and 2021. There were no statistically significant differences between the years. Third, regarding glycemic awareness, in 2017, NERRs had a higher awareness rate than ERRs. There was a trend of increasing awareness in both regions over time. Statistically significant differences were observed only in 2017 and 2021. Fourth, regarding the completion of diabetes management education, NERRs had higher completion rates than ERRs in 2017, 2019, and 2021. However, the difference in completion rates between the two regions decreased from 2.14% in 2017 to 1.76% in 2021. Statistically significant differences were observed across all the years.

## 4 Discussion and public health implications

This study aimed to identify regions based on the LEI and healthcare resources and compare disparities in personal health characteristics and health literacy regarding hypertension and diabetes between regions based on the Andersen healthcare utilization model. The main findings of this study are as follows.

First, we identified 25, 26, and 29 ERRs in 2017, 2019, and 2021, respectively. This is consistent with the results of previous studies showing that regions with a high risk of extinction and low healthcare resources are mainly distributed in non-metropolitan regions, such as Gyeongsang-do, Jeolla-do, and Chungcheong-do (18, 38). This indicates a spatial connection between ERRs and local healthcare resources, and the spatial concentration in non-metropolitan regions must be considered in conjunction with socioeconomic conditions related to decreasing population, weakening economic power, and declining healthcare infrastructure.

Second, the analysis of individual-level demographic characteristics and unmet medical needs showed that ERRs had a higher average age, a higher proportion of people with a spouse, and a higher proportion of economically active people than NERRs. This is consistent with the results of previous studies showing that people in non-metropolitan regions are aging, married, and more economically active (39, 40). Lee (39) emphasized that although metropolitan regions are slowing the aging rate due to population inflow, non-metropolitan regions are becoming polarized by accelerating the aging rate due to population outflow, especially among individuals in their 20s. Park and Park (40) have reported that between 1980 and 2000, the participation rate of rural populations in economic activity was higher than that of the urban population within the same period. This is attributed to the fact that metropolitan regions are known for their focus on the development of tertiary and service industries, thereby providing diverse jobs. In contrast, non-metropolitan regions rely heavily on the agricultural industry, resulting in passive participation in economic activities due to excessive competition from the higher population density in metropolitan regions. However, access to healthcare and education levels resulting from unmet medical needs are lower in ERRs than in NERRs. This indicates a constraint on healthcare access due to the imbalance of healthcare resources between metropolitan and non-metropolitan regions and is consistent with the results of previous studies showing that differences in educational support in metropolitan regions become more polarized over time (18, 41, 42). The presence of unmet medical needs and low education levels in ERRs demonstrates the disparity in healthcare and educational resources, leading to varied environments in metropolitan and non-metropolitan regions.

Third, the results of the analysis of ERRs and NERRs for health literacy in hypertension and diabetes were as follows. The results by region in 2017 and 2019 show that there was no significant difference between the two regions regarding diagnosis and medication. However, there was a difference between the two regions regarding awareness and management education, with ERRs showing a lower level compared to NERRs. Similarly, in 2021, diagnosis and medication use showed a clear difference between the two regions, thereby indicating a lower level of health literacy in ERRs than in NERRs. Next, we evaluated the regional analysis of health literacy related to diabetes and found no significant differences in diagnosis and medication use between the two regions in 2017, 2019, and 2021. Although NERRs had a higher awareness rate than ERRs in 2017, the difference became insignificant in 2019 and 2021. In contrast, there was a clear difference between the two regions in management education over all 3 years, with ERRs showing lower levels than NERRs. Overall, these results are consistent with those of previous studies that have found no significant differences in diagnoses and treatment rates for hypertension and diabetes in regions with decreasing populations, such as rural areas, compared to regions with non-decreasing populations, such as urban areas. Significant differences were also observed in awareness levels for hypertension and diabetes, as well as experience with education on the management of such chronic diseases (43, 44). This indicates that the nationwide COVID-19 outbreak during the first half of 2020 adversely affected access to the diagnosis and treatment of people with hypertension, thereby exacerbating regional disparities (45, 46). Choi et al. (30) found no significant differences in the diagnoses and treatment rates of hypertension and diabetes when comparing the characteristics of key health indicators in regions with decreasing and non-decreasing populations. However, significant differences were observed in the levels of chronic disease awareness and management education experiences. Regarding those who did not receive management education for hypertension, Lee and Lee (43) found a significant difference between rural older adults (75%) and urban older adults (23.4%). Park and Jung (44) also examined the level of health literacy, including education and awareness of disease management, among selected community-based older adults and found significant differences based on where they lived. Additionally, it was found that the COVID-19 pandemic limited routine outpatient visits among hypertensive patients for safety reasons, thereby disrupting access to primary care information, one of the primary goals of hypertension management guidelines (45). A recent study (46) found that participation rates in health checkups decreased across all regions during the COVID-19 pandemic, with significant differences being associated with regional vulnerability factors.

In conclusion, we found that as the number of ERRs increases, the imbalance of healthcare resources between various regions becomes increasingly pronounced. Simultaneously, health disparities between regions become polarized regarding preventive and proactive healthcare. This attribute demonstrates that in metropolitan regions with large populations and high levels of affordability, healthcare resources are expanding to meet increasing healthcare needs; however, in non-metropolitan regions with gradually declining populations and increasing levels of affordability, healthcare resources are declining due to decreased investments in facilities provided by healthcare organizations. Despite these imbalances in healthcare resources, regional disparities in health management, specifically regarding diagnosis and treatment, are not yet profound. This demonstrates that ERR residents can access medical services, such as diagnosis and medication prescriptions, at levels similar to NERR residents. However, awareness and education on blood pressure and blood glucose management for hypertension and diabetes were lower in ERRs compared to those in NERRs, indicating that healthcare services in ERRs are more focused on diagnostic and therapeutic post-treatment rather than preventive and proactive health management. Chronic diseases, such as hypertension and diabetes, can lead to severe health outcomes due to complications. In emergencies, such as cardiovascular complications, hypoglycemia, or hyperglycemia, the lack of readily available healthcare resources makes prevention and management more crucial (47). Therefore, patients in ERRs, where healthcare resources are relatively scarce, need education on recognizing their blood pressure and blood glucose levels and continuous management practices such as exercise and dietary control. In South Korea, approaches to the prevention and management of chronic diseases can be divided between metropolitan and non-metropolitan areas (48). In metropolitan areas, sufficient healthcare resources allow for the operation of health centers in each region, providing residents with easy access to medical services. Conversely, in non-metropolitan areas, limited healthcare resources have necessitated efforts to minimize medical service gaps, such as through mobile health check-up services and the application of digital healthcare (49). Therefore, we propose the following two measures to address the issue of health disparities in ERRs. First, the government should encourage cooperation with private healthcare organizations to provide health education programs aimed at ensuring health management education in vulnerable areas. Considering the concentration of medical institutions in metropolitan regions, there are fewer opportunities for health education provision in rural communities, such as that focusing on chronic disease management. Additionally, a collaborative system between public and private healthcare organizations should be established to reduce the patient burden of activities related to disease prevention, rather than disease treatment. Second, awareness and education regarding chronic disease management should be enhanced through contactless medical and healthcare services, such as those based on digital healthcare and telemedicine. As healthcare resources diminish, accessing preventive healthcare services becomes increasingly challenging for individuals. This can be mitigated by implementing community care services rooted in digital healthcare and telemedicine, which can help provide health education programs that do not necessitate physical patient contact.

This study has several limitations. First, it did not consider all the different types of chronic diseases. When evaluating health literacy for the prevention and management of chronic diseases, it is appropriate to compare other types of chronic diseases, such as hyperlipidemia, cerebrovascular disease, and dementia, in addition to hypertension and diabetes. However, due to data limitations, health literacy was only evaluated and analyzed for two chronic diseases. Second, complex chronic disease groups involving multiple chronic diseases were not considered. Although it is appropriate to categorize different severity levels based on the number of chronic diseases, due to data limitations, health literacy was analyzed only for questions related to hypertension and diabetes. Third, we could not use panel data from the same respondents in each year. To examine trends over time, it is appropriate to use a panel analysis that considers time variation by tracking the same respondents every year. However, there are limitations in generalizing the results of the analysis using only a single year of collected data. Therefore, future studies should consider various chronic diseases, the number of chronic diseases, their severities, and the time variations involved to allow for an increasingly in-depth analysis of the underlying causes.

Despite these limitations, this study has several strengths. First, it utilizes a combination of community-and individual-level data to ensure data diversity. Through individual and community interactions, we can learn about the education, income, and health levels of residents as well as the environmental characteristics of the region. By synthesizing this information, local and national governments can establish policies aimed at revitalizing the society and the economy, while promoting the formulation of healthcare policies that fit the characteristics of various regions. Second, this study can improve the model’s accuracy by considering homogeneity at the individual level, except for the number of healthcare resources and the LEI between the two regions to be compared. The use of a general linear regression model does not consider the interactions within the sample population, which may compromise the model’s accuracy. However, a model that considers propensity score matching reduces the bias between the treatment and control groups, which can improve its accuracy by ensuring the homogeneity of potentially influential factors. Third, a time-series data analysis can reveal local changes and trends over time. Over time, contextual effects at the local level may affect individuals differently, thereby allowing for the prediction of future health behaviors. These results are a crucial reference for improving and supporting policies aimed at improving community health and reducing health inequalities.

## 5 Conclusion

This study differs from previous studies in that it combines community-level data on LEI and healthcare resources with individual-level data on health characteristics and health literacy for hypertension and diabetes. This indicates that healthcare in South Korea is still diagnostic and treatment-oriented, demonstrating the need to shift healthcare processes toward prevention and building health systems based on local vulnerabilities.

Currently, local extinction is a crisis in South Korea. This will further exacerbate population decline, economic stagnation, and reduced access to healthcare services, thereby negatively affecting the health and well-being of local residents. Therefore, government and related organizations must develop strategies aimed at strengthening the healthcare infrastructure and enhancing community cooperation as well as a sense of community. Accordingly, the results of this study can be utilized as a basis for the formulation of policies aimed at addressing regional disparities in chronic disease prevention and management, while ensuring community-level health promotion, with health equity in mind.

## Data availability statement

The original contributions presented in the study are included in the article/Supplementary material, further inquiries can be directed to the corresponding author/s.

## Author contributions

SJ: Conceptualization, Data curation, Investigation, Writing – original draft, Writing – review & editing. YK: Conceptualization, Data curation, Formal analysis, Investigation, Visualization, Writing – original draft, Writing – review & editing. HL: Investigation, Writing – original draft. CS: Investigation, Writing – original draft. MS: Conceptualization, Writing – review & editing. MC: Conceptualization, Supervision, Writing – review & editing.

## References

[1] OECD. Adapting regional policy in Korea: Preparing regions for demographic change, OECD rural studies. Paris: OECD Publishing (2022).

[2] Lee T Shin H Im J Jung S. A study on monitoring medically underserved areas in 2021. Republic of Korea: Ministry of Health and Welfare. (2021).

[3] National Assembly Research Service (2022). NARS issue of the year 2023. Available at: https://www.nars.go.kr/report/view.do?cmsCode=CM0073&brdSeq=41053 (Accessed December 30, 2022)

[4] Lee SH. Seven analyses on regional extinction in Korea. Regional Employment Trend Brief. p. 4–17. (2016). Available at: https://www.keis.or.kr/user/extra/main/2405/publication/reportList/jsp/LayOutPage.do?categoryIdx=129&pubIdx=2237&reportIdx=3362&spage=3 (Accessed March 8, 2016).

[5] Cha M. Age of population decline, Japan’s local regeneration strategies and proposals for regional spatial restructuring. Korea Res Institute Hum Settlements. (2016) 555:1–8.

[6] Lee H. Rural extinction: survival strategies of cities and regions collapsing in chain reactions due to population decline. J Incheon Stud. (2016) 24:217–25.

[7] Kwon Y Sohn M Choi M. Unmet healthcare needs and the local extinction index: an analysis of regional disparities impacting South Korea’s older adults. Front Public Health. (2024) 12:1423108. doi: 10.3389/fpubh.2024.1423108 39148647 PMC11325592

[8] Wishner J Solleveld P Rudowitz R Paradise J Antonisse L. A look at rural hospital closures and implications for access to care: three case studies. Washington, D.C., USA: Kaiser Family Foundation (2016). 3 p.

[9] Reynolds R Dennis S Hasan I Slewa J Chen W Tian D. A systematic review of chronic disease management interventions in primary care. BMC Fam Pract. (2018) 19:11. doi: 10.1186/s12875-017-0692-3 29316889 PMC5759778

[10] Kwon YC Kim KH Chang DM. A study on small area variations of hospital services utilization in some acute diseases-focused on gastric diseases and acute appendicitis. J Digit Converg. (2012) 10:193–200.

[11] Jeong J Kim C Shin M Ryu S Hong J Kim N. Factors related with regional variations of health behaviors and health status: based on community health survey and regional characteristics data. Korea Public Health Res. (2017) 43:91–108.

[12] Nutbeam D Kickbusch I. Health promotion glossary. Health Promot Int. (1998) 13:349–64.

[13] Zhang NJ Terry A McHorney CA. Impact of health literacy on medication adherence: a systematic review and meta-analysis. Ann Pharmacother. (2014) 48:741–51. doi: 10.1177/1060028014526562 24619949

[14] Choi S Kim H. Health literacy status and implications for adults in South Korea. Health Welf Issue Focus. (2021) 413:1–10.

[15] Hersh L Salzman B Snyderman D. Health literacy in primary care practice. Am Fam Physician. (2015) 92:118–24. PMID 26176370 26176370

[16] Mackey LM Doody C Werner EL Fullen B. Self-management skills in chronic disease management: what role does health literacy have? Med Decis Mak. (2016) 36:741–59. doi: 10.1177/0272989X16638330 27053527

[17] Choi S. Analysis of policy instruments for reducing regional healthcare disparities: focusing on the rationality of objectives and means. Korean Assoc Policy Analysis Eval. (2019):197–245.

[18] Ko M Kim K. Analyses on the changes in the spatial distribution of Korean local extinction risk. J Korean Cartogr Assoc. (2021) 21:65–74. doi: 10.16879/jkca.2021.21.1.065

[19] Yun JM Cho YJ. Analysis of changes in the risk of extinction in Haengjeongri unit villages using the local extinction index-a case study on Chungcheongnamdo. J Korean Soc Rural Plan. (2021) 27:103–16.

[20] Yoon B Kim K. A study on the spatial distribution and the actual status of the regional extinction risk in Chungcheongbuk-Do. Korean Urban Geograph Soc. (2022) 25:55–65. doi: 10.21189/JKUGS.25.3.5

[21] Bovet P Gervasoni JP Mkamba M Balampama M Lengeler C Paccaud F. Low utilization of health care services following screening for hypertension in Dar Es Salaam (Tanzania): a prospective population-based study. BMC Public Health. (2008) 8:407. doi: 10.1186/1471-2458-8-407 19087300 PMC2615777

[22] Ahn KS Park SK Cho YC. Risk factors for hypertension of middle aged male workers using data from health check-ups. J Korea Acad-Ind Coop Soc. (2012) 13:4686–93. doi: 10.5762/KAIS.2012.13.10.4686

[23] Oh H Gil E. Prevalence and risk factors of unmet healthcare needs among Korean adults with hypertension. Korean J Adult Nurs. (2017) 29:22–31. doi: 10.7475/kjan.2017.29.1.22

[24] Yun JM. Study on applicability of village extinction index through comparative study with regional extinction index. J Korean Soc Rural Plann. (2024) 30:1–13. doi: 10.7851/ksrp.2024.30.1.001

[25] An S Kim N Kim Y. Comparison of health status and the effectiveness of health cost between rural and urban residents. (2019). Policy research report Naju. Korea Rural Economic Institute, 257.

[26] Kang YW Ko YS Kim YJ Sung KM Kim HJ Choi HY. Korea community health survey data profiles. Osong Public Health Res Perspect. (2015) 6:211–7. doi: 10.1016/j.phrp.2015.05.003, PMID: 26430619 PMC4551141

[27] Yoon MS Jeong HS Bae BY Hong NY Yim HW. Changes in daily physical activities by income level according to the prevalence of hypertension and diabetes during the COVID-19 pandemic: the 2020 community health survey. Korean J Health Educ Promot. (2022) 39:15–25. doi: 10.14367/kjhep.2022.39.2.15

[28] Phillips KA Morrison KR Andersen R Aday LA. Understanding the context of healthcare utilization: assessing environmental and provider-related variables in the behavioral model of utilization. Health Serv Res. (1998) 33:571. 9685123 PMC1070277

[29] Von Wagner C Steptoe A Wolf MS Wardle J. Health literacy and health actions: a review and a framework from health psychology. Health Educ Behav. (2009) 36:860–77. doi: 10.1177/1090198108322819, PMID: 18728119

[30] Choi SK Kim H Hwang J Chae S Han G Yu J. A study for improving health literacy. Sejong: Korea Institute for Health and Social Affairs. (2020): 47.

[31] Anselin L. “Spatial dependence in linear regression models with an introduction to spatial econometrics.” Handbook of Applied Economic Statistics/Marcel Dekker. (1998) 237.

[32] Moran PA. Notes on continuous stochastic phenomena. Biometrika. (1950) 37:17–23. doi: 10.1093/biomet/37.1-2.17, PMID: 15420245

[33] Tu J Xia ZG. Examining spatially varying relationships between land use and water quality using geographically weighted regression I: model design and evaluation. Sci Total Environ. (2008) 407:358–78. doi: 10.1016/j.scitotenv.2008.09.031, PMID: 18976797

[34] Bertoni D Curzi D Aletti G Olper A. Estimating the effects of Agri-environmental measures using difference-in-difference coarsened exact matching. Food Policy. (2020) 90:101790. doi: 10.1016/j.foodpol.2019.101790

[35] Cousineau M Verter V Murphy SA Pineau J. Estimating causal effects with optimization-based methods: a review and empirical comparison. Eur J Oper Res. (2023) 304:367–80. doi: 10.1016/j.ejor.2022.01.046 PMC12657020 41311879

[36] Stephens DA Nobre WS Moodie EE Schmidt AM. Causal inference under misspecification: adjustment based on the propensity score [with discussion]. Bayesian Anal. (2023) 18:639–94. doi: 10.1214/22-BA1322

[37] Evans SZ. Propensity score matching. Encycl Res Methods Criminol Criminal Justice. (2021) 2:859–64. doi: 10.1002/9781119111931.ch166

[38] Lee HJ Oh JH Kim JH Lee KS. Relationship between local extinction index and medical service uses of chronic diseases. Health Policy Manag. (2021):301–11. doi: 10.4332/KJHPA.2021.31.3.301

[39] Lee T. The impact of regional population mobility on aging [KIRI report]. Korea Insurance Research Institute, (2022) 539; p. 8–15.

[40] Park D Park K. Trend and prospect of rural community change: centered around the change of population structure. Korea Rural Econ Institute. (2003):184. doi: 10.22004/ag.econ.288013

[41] Lee DH. A study on the educational bipolarization between urban area and rural area. Korean J Sociol Educ. (2011) 21:121–48. doi: 10.32465/ksocio.2011.21.2.005

[42] Choi HS Song GJ Ko YS Chae SH Ahn YJ. Status of major health indicators in population shrinking regions. Korea KDCA. (2022) 15:816–28.

[43] Lee JA Lee YN. Comparison of healthy life style and chronic disease management between urban and rural older adults. Korean J Rehabil Nurs. (2012) 15:100–8. doi: 10.7587/kjrehn.2012.100

[44] Park E Jung Y. The association of medical service and medication use information literacy with multi-morbidity. Health Soc Welfare Rev. (2020) 40:222–43.

[45] Ferdinand KC Vo TN Echols MR. State-of-the-art review: hypertension practice guidelines in the era of COVID-19. Am J Prev Cardiol. (2020) 2:100038. doi: 10.1016/j.ajpc.2020.100038 32835351 PMC7361040

[46] Kim Y Park J Park JH. Regional differences in health screening participation between before and during COVID-19 pandemic. Environ Health Prev Med. (2023) 28:8. doi: 10.1265/ehpm.22-00239 36697026 PMC9884562

[47] Silbert R Salcido-Montenegro A Rodriguez-Gutierrez R Katabi A McCoy RG. Hypoglycemia among patients with type 2 diabetes: epidemiology, risk factors, and prevention strategies. Curr Diab Rep. (2018) 18:53. doi: 10.1007/s11892-018-1018-0, PMID: 29931579 PMC6117835

[48] Eun SJ. Trends and disparities in avoidable, treatable, and preventable mortalities in South Korea, 2001-2020: comparison of capital and non-capital areas. Epidemiol Health. (2022) 44:44. doi: 10.4178/epih.e2022067, PMID: 35989656 PMC9754920

[49] Yao R Zhang W Evans R Cao G Rui T Shen L. Inequities in health care services caused by the adoption of digital health technologies: scoping review. J Med Internet Res. (2022) 24:e34144. doi: 10.2196/34144 35311682 PMC8981004

