# Supplementary material for: Regional disparities in health literacy for chronic diseases: focusing on healthcare resources and local extinction index
Source: Front Public Health. 2024 Sep 12;12:1423645. doi: 10.3389/fpubh.2024.1423645 (PMC11428103; doi:10.3389/fpubh.2024.1423645)
Supplement: Supplementary file 1 [file Table_1.docx]

Supplementary Material

**Appendix:**

**Table A1. Propensity score distribution before and after matching**

|  | **2017** | **2019** | **2021** |
| --- | --- | --- | --- |
| **Before** |  |  |  |
| **After** |  |  |  |
