# Supplementary material for: Regional disparities in health literacy for chronic diseases: focusing on healthcare resources and local extinction index
Source: Front Public Health. 2024 Sep 12;12:1423645. doi: 10.3389/fpubh.2024.1423645 (PMC11428103; doi:10.3389/fpubh.2024.1423645)
Supplement: Supplementary file 2 [file Table_2.docx]

Supplementary Material

**Appendix:**

**Table A2. Treatment and control area by years**

| **2017** | | **2019** | | **2021** | |
| --- | --- | --- | --- | --- | --- |
| **Control** | **Treatment** | **Control** | **Treatment** | **Control** | **Treatment** |
| Bupyeonggu | Andongsi | Gangseogu | Andongsi | Gangseogu | Andongsi |
| Gimposi | Bonghwagun | Gimposi | Bonghwagun | Gimposi | Bonghwagun |
| Gyeyanggu | Boryeongsi | Gyeyanggu | Boryeongsi | Gyeyanggu | Boryeongsi |
| Hwaseongsi | Cheongyanggun | Songpagu | Cheongyanggun | Hanamsi | Cheongyanggun |
| Songpagu | Gangneungsi |  | Danyanggun | Junggu | Danyanggun |
|  | Geochanggun |  | Gangneungsi | Seochogu | Gangneungsi |
|  | Hamyanggun |  | Geochanggun | Seongnamsi | Geochanggun |
|  | Hapcheongun |  | Hamyanggun | Songpagu | Hamyanggun |
|  | Hongcheongun |  | Hapcheongun |  | Hapcheongun |
|  | Imsilgun |  | Hongcheongun |  | Hongcheongun |
|  | Jangsugun |  | Imsilgun |  | Imsilgun |
|  | Jeongseongun |  | Jangsugun |  | Jangsugun |
|  | Jinangun |  | Jeongseongun |  | Jeongeupsi |
|  | Mujugun |  | Jinangun |  | Jeongseongun |
|  | Mungyeongsi |  | Mujugun |  | Jinangun |
|  | Namwonsi |  | Mungyeongsi |  | Mujugun |
|  | Pyeongchanggun |  | Namwonsi |  | Mungyeongsi |
|  | Samcheoksi |  | Pyeongchanggun |  | Namwonsi |
|  | Sangjusi |  | Samcheoksi |  | Pyeongchanggun |
|  | Sunchanggun |  | Sangjusi |  | Samcheoksi |
|  | Taebaeksi |  | Sunchanggun |  | Sangjusi |
|  | Uljingun |  | Taebaeksi |  | Sunchanggun |
|  | Yeongdonggun |  | Uljingun |  | Taebaeksi |
|  | Yeongjusi |  | Yeongdonggun |  | Uiseonggun |
|  | Yeongyanggun |  | Yeongjusi |  | Uljingun |
|  |  |  | Yeongyanggun |  | Yecheongun |
|  |  |  |  |  | Yeongdonggun |
|  |  |  |  |  | Yeongjusi |
|  |  |  |  |  | Yeongyanggun |
